# Complete genome sequence of deformed wing virus isolated from honeybees (*Apis mellifera* L.) in Iraq

**DOI:** 10.1128/mra.00770-24

**Published:** 2024-10-14

**Authors:** Ali Sadiq, Osamah Alisawi, Mohammed Shakir Mansor

**Affiliations:** 1 Plant Protection Department, Faculty of Agriculture, University of Tikrit, Tikrit, Iraq; 2 Plant Protection Department, Faculty of Agriculture, University of Kufa, Najaf, Iraq; Portland State University, Portland, Oregon, USA

**Keywords:** bioinformatics, deformed wing virus, honeybee genome, RNA-seq

## Abstract

Here, we report the complete genome sequence of deformed wing virus (DWV) isolated from honeybees (*Apis mellifera* L.) in Iraq, named the Tikrit isolate. The phylogeny revealed that the Tikrit isolate has a close relationship to the Iraqi isolate DWV-Iraq-2023 and related to isolates from France, the United Kingdom, and Israel.

## ANNOUNCEMENT

**Fig 1:**
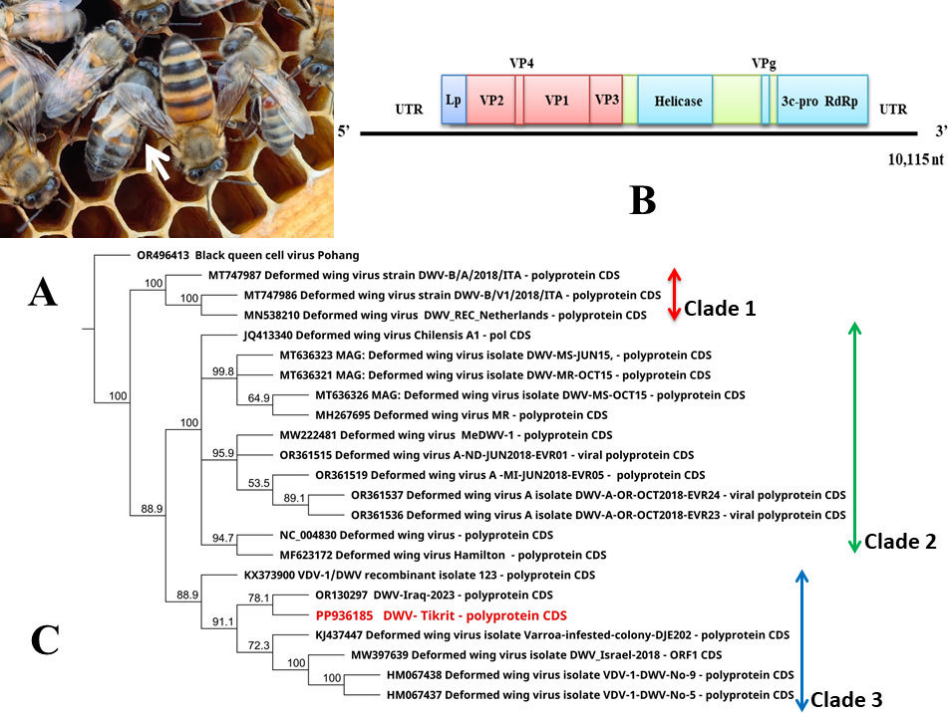
Newly emerged bees with deformed wings usually associated with the symptomatic deformed wing virus (the honeybee indicated in a white arrow) (**A**). Genome organization of DWV shows the major structural proteins, VP1, VP2, and VP3, in addition to helicase, 3c-pro, and RdRp (**B**). A phylogeny tree for DWV indicates that the Tikrit isolate is closely related to the Iraqi isolate DWV-Iraq-2023 (OR130297) and related to isolates from France, the United Kingdom, and Israel within clade 3. Geneious tree builder v. 2024 was used to build the tree, and ClustalW was used to align the whole-genome nucleotide sequences. The best substitution model Hasegawa–Kishino–Yano (HKY) was applied to build the neighbor joining tree, and 1,000 bootstraps were used to infer the tree. The outgroup member was Black queen cell virus Pohang (**C**).

Deformed wing virus (DWV) from the family Iflaviridae, among multiple viruses, infects honeybees (*Apis mellifera* L.) and causes colony collapse (1). There has been tremendous interest in and the study of deformed wing virus (DWV), a widespread, widely studied insect pathogen. A new vector, the ectoparasitic mite *Varroa destructor*, has dramatically altered DWV epidemiology since DWV was previously present in honeybees. Over 32 countries, at least 55% of colonies/apiaries are infected with DWV (2). Iflaviridae viruses, which are members of the order Picornavirales, have nonenveloped icosahedral virions with a diameter of about 30 nm (3). Capsids of iflaviruses protect ssRNA genomes of 10,000 nt that are translated into polyproteins that are cleaved cotranslationally and posttranslationally by viral proteases (4). Information on the virus presence in Iraq is limited to date. The whole body of a honey bee with deformed wing symptoms was taken from a collapsed colony in the Tikrit region, Saladin Province, on 12 Nov 2023 and immersed in 5 x volume of manually prepared RNAlater (2 mL) in a single Eppendorf tube (Fig. 1A). RNA was extracted from the honeybee head tissue sample using the RNeasy Mini Kit (QIAGEN, Hilden, Germany) following the manufacturer’s instructions. A total RNA library prep kit from TruSeq RNA Prep v2 was used to prepare the sequencing library, and it was subjected to whole-genome sequencing (Platform: NovaSeq 6000; Application: WGS, Nano350, mRNA), and the reads were then trimmed using Trimmomatic-0.39 and BBduk v 37.22 in Geneious Prime 2024.0.5 (5). Geneious Prime was applied to map the clean and paired-end reads against the reference genome of the deformed wing virus using the Geneious RNA mapper (sensitivity: medium–low). The consensus sequence was extracted and aligned in pairwise alignment with the reference genome (OR130297) to obtain the complete genome sequence and then analyzed with NCBI-BLASTx v.5 (6) and Open Reading Frame Finder in Geneious Prime 2024.0.5. Unless otherwise specified, all tools were run with default parameters. Total honeybee RNA-seq was 73,431,574 paired-end reads of 101 bases with 40.4% GC content were obtained from the RNAseq library. Based on mapping to the reference tool in Geneious prime, the mapped reads were 7,655,841. After aligning and trimming the consensus sequence (10,288 bases), the complete genome for the Tikrit isolate of the virus was 10,115 bases long with 39.7% GC content. The sequence begins with a 1,110 nt nontranslated leader sequence, followed by an open-reading frame of 8,682 nt encoding a polyprotein with 2,894 residues, followed by a 323-nt non-translated region and a poly (A) tail. VP1, VP2, and VP3 were identified as the three major structural proteins in addition to helicase, 3c-pro, and RNA-dependent RNA polymerase (RdRp) (Fig. 1B). The DWV-Tikrit sequence shows the most nucleotide similarity with OR130297 (Iraq) (97.7%), KX373900 (France) (97.3%), KJ437447 (United Kingdom) (97.4%), MW397639 (Israel) (97.1%), HM067438 (96.7%), and HM067437 (95.7%) (United Kingdom). Additionally, the phylogeny confirmed the isolate identities by placing the closest isolates in the same clade (Fig. 1C).

## Data Availability

The total RNA sequence of the honeybee has been deposited in GenBank under the accession number SRR29768430 . The complete sequence of deformed wing virus deposited in GenBank under accession number PP936185.1.
